# You Spin Me Right Round: Cross-Relationship Variability in Interpersonal Emotion Regulation

**DOI:** 10.3389/fpsyg.2012.00394

**Published:** 2012-10-08

**Authors:** Karen Niven, Ian Macdonald, David Holman

**Affiliations:** ^1^Manchester Business School, University of ManchesterManchester, UK; ^2^Department of Psychology, University of SheffieldSheffield, UK

**Keywords:** interpersonal emotion regulation, emotion regulation, interpersonal behavior, spin, relationships

## Abstract

Individuals use a range of interpersonal emotion regulation strategies to influence the feelings of others, e.g., friends, family members, romantic partners, work colleagues. But little is known about whether people vary their strategy use across these different relational contexts. We characterize and measure this variability as “spin,” i.e., the extent of dispersion in a person’s interpersonal emotion regulation strategy use across different relationships, and focus on two key questions. First, is spin adaptive or maladaptive with regard to personal well-being and relationship quality? Second, do personality traits that are considered important for interpersonal functioning (i.e., empathy, attachment style) predict spin? The data used in this study is drawn from a large online survey. A key contribution of this study is to reveal that people who varied the type of strategies they used across relationships (i.e., those with high spin) had lower positive mood, higher emotional exhaustion, and less close relationships. A further key contribution is to show that spin was associated with low empathic concern and perspective taking and high anxious attachment style. High variability in interpersonal emotion regulation strategies across relationships therefore appears to be maladaptive both personally and socially.

## Introduction

People often try to shape the feelings of others in interpersonal relationships. This is reflected in the many anecdotal tales of people cheering friends up, making family members feel guilty, calming anxious coworkers, or making romantic partners feel jealous. Attempting to influence the feelings of a relationship partner has been termed “interpersonal emotion regulation” and research has documented that people use a broad range of interpersonal emotion regulation strategies (Niven et al., 2009). Furthermore, the choice of which strategies to use can have important consequences for the well-being of both parties involved (those who engage in the attempts and those they are directed toward) as well as the quality of the relationship between them [e.g., Niven et al. (2012a)]. What is less clear, however, are the implications of using the same or different strategies across various relationships. Varying one’s strategy use across relationships could signal an attempt to match strategy choice to the relational situation and thus be considered functional. However, it could also be a sign of an underlying instability and be perceived by relationship partners as inconsistent and thus be considered dysfunctional.

The first aim of this paper is to examine whether it is adaptive or maladaptive to have higher variation in the use of interpersonal emotion regulation strategies across different types of relationship (romantic, friendly or familial, work), focusing on the outcomes of personal well-being (i.e., positive mood, emotional exhaustion) and relationship quality (i.e., relational closeness). Based on analytic innovations within the psychology of interpersonal behavior (Moskowitz and Zuroff, 2004), we characterize and measure variability in interpersonal emotion regulation strategy use across different relationships as a form of interpersonal “spin,” i.e., the extent of dispersion in a person’s interpersonal behavior across different social contexts. Because interpersonal spin may have important consequences for people’s well-being and relationships, it is also important to know whether certain individuals are more prone to interpersonal spin than others. The second aim of the paper is therefore to examine whether personality traits considered important for interpersonal functioning (i.e., empathy, attachment style) are antecedents of spin.

Emotion regulation refers to “the process of initiating, maintaining, modulating, or changing the occurrence, intensity, or duration of internal feeling states” (Eisenberg et al., 2000, p. 137). Research on this process has traditionally focused on the ways that people try to manage and control their own emotions (intrapersonal emotion regulation), for example, distinguishing different types of strategies people use to shape their feelings (Gross, 1998; Parkinson and Totterdell, 1999) and investigating their relative effectiveness (Augustine and Hemenover, 2009; Webb et al., 2012).

Increasingly, however, researchers are interested in the social aspects of emotion regulation. Many theoretical models begin with the basic assumption that emotions and emotion regulation are typically experienced and engaged in the presence of others (e.g., Côté, 2005; Hareli and Rafaeli, 2008; Van Kleef, 2009), and it is now well-established that even when we are alone, our attempts to manage our emotions may be in anticipation of social interaction (Erber et al., 1996).

Within this broader context, the process of interpersonal emotion regulation has emerged as an important research concern. Interpersonal emotion regulation concerns deliberate attempts to influence others’ feelings. Although interpersonal emotion regulation can be used by larger social groups (e.g., a support group working together to alleviate the negative emotions of one of its members; Thoits, 1996) or directed toward multiple people (e.g., a sports coach trying to motivate and enthuse members of a team; Friesen et al., 2011), in this paper we focus on interpersonal emotion regulation in which one person (known as the “agent”) attempts to shape the feelings of another person (the “target”). Dyadic interpersonal emotion regulation attempts have been reported in a broad range of social relationships, including romantic relationships (Vangelisti et al., 1991), familial relationships (Thompson and Meyer, 2007), friendships (Nils and Rimé, 2012), and work relationships (Rafaeli and Sutton, 1991; Locke, 1996; Francis et al., 1999; Pierce, 1999; Lively, 2000).

A person engaging in interpersonal emotion regulation has many strategies at his or her disposal. A classification developed by Niven et al. (2009) highlighted two main distinctions between strategy types. The first distinction concerns whether the regulatory motive behind the strategy is to improve how the target feels or to worsen the target’s feelings. The second distinction concerns whether the strategy is implemented using cognitive or behavioral resources. Cognitive strategies involve the agent trying to influence a target’s thoughts about his or her feelings or situation, e.g., an agent reinterpreting a situation to make a target feel better. Behavioral strategies involve the agent using his or her behavior to change the target’s feelings, e.g., an agent sulking to make a target feel worse. Thus, their classification proposes four key strategy types: cognitive improving, behavioral improving, cognitive worsening, and behavioral worsening (see Table 1 for example strategies).

**Table 1:** **Interpersonal emotion regulation strategy types**.

		Regulatory motive	
		To improve affect	To worsen affect
**Implementation resource**	**Cognitive**	Engaging with the target’s cognitions about his or her feelings or a situation in order to improve his or her affect, *e.g., giving the target advice*	Engaging with the target’s cognitions about his or her feelings or a situation in order to worsen his or her affect, *e.g., complaining about the target’s behavior*
	**Behavioral**	Pleasant behaviors intended to improve the target’s affect, *e.g., spending time with the target*	Unpleasant behaviors intended to worsen the target’s affect, *e.g., being rude to the target*

Initial studies exploring the relative effects of these strategy types have primarily concentrated on differences between improving and worsening strategies. Improving strategies have been found to have positive consequences for the short-term affect and longer-term well-being of the agent and target of regulation and the quality of the relationship between the two, while worsening strategies are found to have negative consequences for these outcomes (Niven et al., 2007, 2012a,b). A recent study by Nils and Rimé (2012), however, noted divergent consequences of improving strategies that engaged cognitively (labeled by the authors as “agentic” strategies) and those that focused on more behavioral means of regulation (labeled as “communal”). Broadly, cognitive improving strategies facilitated greater emotional recovery from emotional events, whereas behavioral improving strategies had more positive social consequences, including feelings of proximity between agent and target.

While the emerging body of research concerning interpersonal emotion regulation has much to say about the use and effects of different strategies within social relationships, little is known about whether people vary their use of interpersonal emotion regulation across social contexts and if it is adaptive or maladaptive to do so. In the present study, we explore this question by investigating whether high variation in one’s use of interpersonal emotion regulation across relationships (i) facilitates or inhibits personal and social functioning, and (ii) is associated with personality traits that are typically considered functional or dysfunctional for interpersonal relationships. We focus on the use of interpersonal emotion regulation within three distinct types of relationships: romantic, familial or friendly, and work. According to Neyer et al. (2011), relationship types can largely be differentiated based on their degree of emotional closeness (defined as a sense of kinship with others) and reciprocity (defined as norms regarding equity, balance, and fairness). By selecting the three relationships of interest in our research, we capture a high closeness-high reciprocity relationship type (romantic), a high closeness-low reciprocity relationship type (familial or friendly), and a low closeness-high reciprocity relationship type (work), thus providing a good range of relationships to study variability across.

The idea that people might vary their behavior across different situations has been studied for some years now by researchers of interpersonal behavior (see Moskowitz, 2009, for a review). Critiquing the view popularized by personality researchers that interpersonal behavior is necessarily consistent, such researchers have investigated the extent to which people vary their behavior across time and situations. Drawing on the interpersonal circumplex model (Wiggins, 1991), research in this area differentiates interpersonal behaviors according to two key dimensions: communality (is the behavior agreeable or quarrelsome); and agency (is the behavior dominant or submissive). Studies investigating the extent to which these types of behaviors are used in different situations have reported links between variability and stable personality traits, such as extraversion and neuroticism (Moskowitz and Zuroff, 2004) as well as links with important outcomes including well-being and the development of high-quality relationships (e.g., Erickson et al., 2009; Côté et al., 2011).

Although early studies of variability focused on taking measures in multiple situations and calculating the standard deviation or coefficient of variability of mean scores across the various situations as an index of variation (e.g., Fleeson, 2001), the now-dominant method used to operationalize variability in interpersonal behavior was proposed by Moskowitz and Zuroff (2004). Like earlier indices, Moskowitz and Zuroff’s method involves collecting data about people’s engagement with interpersonal behavior across different situations. However, rather than calculating the variability of either grand mean scores of the focal process (e.g., variability in the total amount of interpersonal behavior used) or calculating separate indices of variability for each facet of interest (e.g., variability in agreeable, quarrelsome, dominant, and submissive behaviors), this method allows researchers to take into account variability in the distinct dimensions within a single score. The popularity of this method is such that it is now being applied to studying variability in other processes, including “core affect” (i.e., people’s background feeling states), in which researchers are concerned both valence (is the state pleasant or unpleasant) and arousal (is the state highly activated or deactivated; Kuppens et al., 2007). Certainly, the advantage of this method for the current study is clear, as interpersonal emotion regulation, like interpersonal behavior and core affect, is not a unidimensional construct. Rather, research has clearly established two key dimensions along which interpersonal emotion regulation strategies differ (motives and resources; Niven et al., 2009).

**Figure 1:**
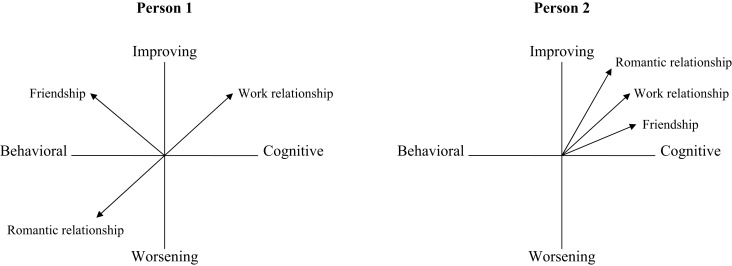
**Illustration of a person with high spin (left panel) and low spin (right panel)**.

Applied to the present study, Moskowitz and Zuroff’s (2004) method is used to quantify the amount of variability that a person displays in the overall nature of interpersonal emotion regulation (taking into account both the motives and resources involved) across all relationships of interest. The single variability score produced by this method, referred to as “spin,” reflects the extent of dispersion in a person’s strategy use across social relationships. A demonstration of high and low spin is illustrated in Figure 1. The two dimensions that characterize interpersonal emotion regulation strategies are plotted such that each vector in the figure represents the overall nature of strategy use within a given relationship; a person’s motive for regulation (calculated by subtracting the extent to which a person uses strategies to worsen emotions within a given relationship from the extent to which a person uses strategies to improve emotions within that relationship) is plotted along the vertical axis, while his or her resource (calculated by subtracting behavioral strategies from cognitive strategies) is plotted along the horizontal axis. It should be noted, however, that a person’s spin score is independent of the axes, such that a person would have the same level of spin if motive was represented along the horizontal axis and resource along the vertical axis. Person 1, shown in the left panel, has high spin; in his or her work relationship cognitive improving strategies are favored, in the friendship behavioral improving strategies are used, while in the romantic relationship behavioral worsening strategies are preferred. In contrast, Person 2, shown in the right panel, exhibits low spin; there is consistency within all of his or her relationships, with mostly cognitive improving strategies used.

Theoretically, there are reasons to believe that high variability in the use of interpersonal emotion regulation strategies might be adaptive. In different relationships there are likely to be different demands and social norms, and it would seem important to display a certain degree of flexibility in the way one attempts to regulate a relationship partner’s emotions (the functional flexibility argument; Paulhus and Martin, 1988). Certainly, research concerning interpersonal emotion regulation highlights situational differences with respect to the appropriateness and effectiveness of particular strategies. For example, Francis et al.’s (1999) research in hospitals highlights how “dark” humor can be appropriate as a way for medical professionals to improve the feelings of a coworker but not a patient.

However, there are also reasons to believe that high variability might be maladaptive. It has been suggested that high variability is the result of heightened reactivity to the influence of situations, such that the person is unable to maintain consistency and to develop effective strategies for interaction (Erickson et al., 2009). This may cause difficulties with regard to social relationships, as people tend to prefer consistency in their interaction partners because it helps them to build a mental model of who the person is and how to interact with them (Devine et al., 1989). As such, high variability may be unhelpful for the development of close bonds, and may impact negatively on perceptions of relationship closeness, i.e., the extent of overlap between another person’s life and one’s own (Aron et al., 1992).

In addition, variability might compromise people’s well-being. More inconsistent interactions are likely to be more demanding to carry out and will thus require more attention and effort, particularly if the person needs to repair interactions that have gone wrong (Schegloff et al., 1977). Increased attention and effort are in turn likely to induce heightened physiological activation (Dormann and Zapf, 2004) and overtax personal energy resources (Hobfoll, 1989). Difficult interactions may also make it less likely that a person will attain their goals which, according to goal-based theories of behavior, will negatively impact on the personal resources of self-competence and self-efficacy (Locke and Latham, 1990; Bandura, 1997). Both depletion of energy resources and threats to personal resources are likely to lead to increased feelings of emotional exhaustion – a state of emotional depletion and fatigue – and decreased positive mood (Hobfoll, 1989). In sum, there are strong theoretical reasons to expect high interpersonal spin to have maladaptive outcomes with regard to relationship quality and psychological well-being.

Although both perspectives are equally viable theoretically, the cumulative evidence from studies of spin in interpersonal behavior provides strong support for the perspective that high variability is maladaptive. Higher levels of interpersonal spin have been linked to indicators of poor quality relationships, including low relationship closeness, low dyadic adjustment, and high coworker social avoidance (Côté et al., 2011), and poor well-being, including depression and distress (Erickson et al., 2009). Studies of affect spin similarly report higher variability to be associated with poorer psychological adjustment (Kuppens et al., 2007). We therefore expect spin to be negatively associated with relationship closeness and positive mood and to be positively associated with emotional exhaustion.

Given that interpersonal spin might be maladaptive, it would seem important to understand its antecedents. Previous research has revealed that interpersonal spin is positively associated with personality traits typically considered to be dysfunctional (e.g., neuroticism) and negatively associated with functional traits (e.g., agreeableness, extraversion, self-esteem; Moskowitz and Zuroff, 2004; Côté et al., 2011). In addition, people with borderline personality disorder exhibit significantly higher spin in interpersonal behavior compared to non-clinical control participants (Russell et al., 2007). According to Moskowitz and Zuroff (2004), these findings are indicative of spin reflecting behavioral lability, i.e., variability that is poorly controlled, as opposed to behavioral flexibility, which is variability that stems from effective responses to different situations. In the present study, we build on this research by exploring the links between interpersonal emotion regulation spin and two sets of personality traits, one set that is typically considered functional for interpersonal relationships (empathic concern and perspective taking) and one set that is typically considered dysfunctional (avoidant and anxious attachment styles).

Empathic concern and perspective taking are two facets of empathy, i.e., “the reactions of one individual to the observed experiences of another” (Davis, 1983, p. 113). Empathic concern refers to feelings of sympathy or concern for others and is the main emotional aspect of empathy, while perspective taking refers to the tendency to adopt the point of view of others and is the main cognitive aspect. Empathy is thought to be a highly functional trait for the development of high-quality connections with others, and both empathic concern and perspective taking have been associated with improved social functioning in past research (e.g., Oswald, 1996; Litvack-Miller et al., 1997). Attachment styles are “systematic patterns of relational expectations, emotions, and behaviors that result from internalization of a particular history of attachment experiences” (Mikulincer and Shaver, 2005, p. 150). An avoidant attachment style is characterized by a distrust of relationship partners’ goodwill and the need to maintain independence and emotional distance, whereas an anxious attachment style is characterized by worrying that relationship partners will not be available in times of need, a strong need for closeness, and a fear of rejection (Brennan et al., 1998). Both forms of attachment are thought to be highly dysfunctional for the development of relationships as they increase anger episodes and depression, and reduce compassion and caregiving behaviors, all of which may drive potential relationship partners away (Mikulincer, 1998; Mikulincer et al., 2005; Shaver et al., 2005).

Based on the existing evidence that spin tends to be maladaptive for the development of close relationships (Côté et al., 2011), and that it is reflective of behavioral lability (Moskowitz and Zuroff, 2004), it seems likely that those people who display higher variability in interpersonal emotion regulation will have more dysfunctional traits. We therefore expect empathic concern and perspective taking to be negatively associated with interpersonal spin and avoidant and anxious attachment to be positively associated with interpersonal spin.

## Materials and Methods

### Design

A repeated measures study design was used, whereby participants reported their use of interpersonal emotion regulation strategies within up to three specific relationships: a romantic relationship, a work relationship, and a familial relationship or friendship. Those participants who did not have a romantic partner or who did not work did not complete the measures of their interpersonal emotion regulation within those particular relationships. Participants were randomly assigned to complete the interpersonal emotion regulation measures corresponding to each relationship in different orders, and independent-samples analyses of variance (ANOVAs) using the mean interpersonal emotion regulation strategy scores in the three different relationships as dependent variables confirmed no order effects (*F*s < 2.28, *p*s > 0.10). Ethical approval was obtained for the study from the Institute of Work Psychology Research Ethics Committee at the University of Sheffield in the UK (the institution where the first author formerly worked).

### Sample

An online survey was advertised to members of the public via several means, including advertising on websites that promote social sciences research studies and specialist websites designed to target harder-to-reach populations in order to ensure the sample was representative (e.g., lesbian gay bisexual transgender websites), as well as emails to staff and students at several UK universities. To be eligible to take part, people had to be over the age of 16. Informed consent was obtained from all respondents in a form at the start of the survey. A total of 1509 people completed the survey. Because calculating spin requires measures across multiple situations, respondents who only completed a single measure of interpersonal emotion regulation (*N* = 248) were excluded from subsequent analyses, leaving 1261 respondents. A further 50 respondents had to be excluded as their answer pattern for at least one relationship resulted in an overall position at the origin (0,0) and so did not allow calculation of a vector from which spin could be derived (this answer pattern was typically due to respondents giving the same answer to all items in the interpersonal emotion regulation measure).

Our final sample therefore comprised 1211 participants (79% females). The ages of participants ranged from 16 to 71 (*M* age = 30.96 years, SD = 12.08). Of the total sample, 970 participants worked (64% full-time). The largest occupational grouping was professional occupations (*N* = 280), followed by administrative or secretarial occupations (*N* = 149), and manager or senior official (*N* = 74). Students made up the majority of the non-working sample, but there were also 36 unemployed respondents and 4 retired respondents. 55% of respondents completed the survey in their home, and the remainder in their place of work. In total, 663 participants reported on all three relationship types, while the remaining 548 reported on two of the three types. All of the respondents completed the friend or family measure of interpersonal emotion regulation, while 973 completed the romantic partner measure, and 901 completed the work measure.

Due to the relatively high load placed on participants of responding to the interpersonal emotion regulation measure up to three times relating to different relationships, we split our participants randomly into groups to complete our individual difference measures, so that each participant only had to complete one set of measures. A total of 228 participants (79% females; *M* age = 30.09 years, SD = 12.69) provided data about their empathy, and 273 (77% females; *M* age = 31.31 years, SD = 11.65) provided data about their attachment style. The remaining 710 participants completed measures not relevant to the focus of this study, e.g., self-efficacy, emotional expressivity.

### Measures

#### Interpersonal emotion regulation spin

The 12-item extrinsic subscale of the Emotion Regulation of Others and Self (EROS; Niven et al., 2011) measure was used to assess respondents’ use of interpersonal emotion regulation strategies within each relationship. The subscale comprises four factors relating to the four distinct types of interpersonal emotion regulation strategies proposed in Niven et al.’s (2009) classification, each of which is assessed using three items. The scale has been shown to be reliable and valid in previous research (e.g., Niven et al., 2011). To complete the measures, participants were first instructed to bring a particular person to mind (their romantic partner, a friend or relative, or someone they worked with, depending on the relationship in question), and then to indicate the extent to which they had used the various interpersonal emotion regulation strategies to influence the way that person had felt over the previous 4 weeks. The cognitive improving factor was measuring using items such as “I gave [*person x*] helpful advice to try to improve how they felt” (αs for the different relationships ranged between 0.79 and 0.88). An example behavioral improving item was “I did something nice with [*person x*] to try to make them feel better” (αs for the different relationships ranged between 0.81 and 0.85). Cognitive worsening items included “I explained to [*person x*] how they had hurt myself or others, to try to make them feel worse” (αs for the different relationships ranged between 0.76 and 0.82). Finally, the behavioral worsening factor included items such as “I was unfriendly to [*person x*] to try to make them feel worse” (αs for the different relationships ranged between 0.79 and 0.85).

Respondents’ self-reports of their use of interpersonal emotion regulation were validated using a follow-up measure of their strategy use as reported by the other person in each of their relationships. At the end of the survey, respondents were invited to leave the email addresses of those individuals who they had reported their use of interpersonal emotion regulation toward. These people were then contacted with a link to a new survey which comprised a single interpersonal emotion regulation scale; this time people were asked to report on use of strategies by their relationship partner (the original participant) toward themselves over the same 4 week period. Although only a small number of matched pairs were collected (*N* = 50), analyses revealed medium to large sized correlations between original participants’ self-reports of their use of interpersonal emotion regulation strategies and their relationship partners’ reports (cognitive improving *r* = 0.32, *p* < 0.05; behavioral improving *r* = 0.44, *p* < 0.01; cognitive worsening *r* = 0.46, *p* < 0.01; behavioral worsening *r* = 0.64, *p* < 0.01), providing support for the validity of our data.

**Figure 2:**
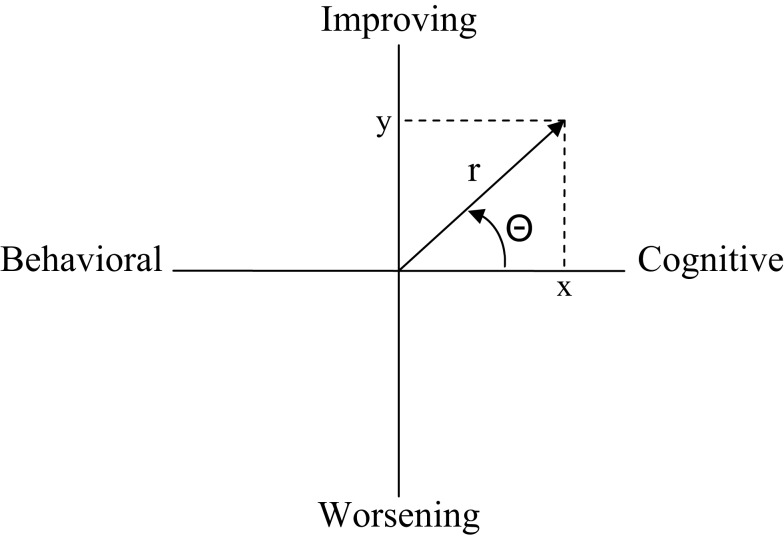
**Illustration of Cartesian (*x*, *y*) and polar (*r*, Θ) coordinates used to calculate spin**.

The self-report data was used to calculate spin. The first step to calculate spin was to create a motive score and a resource score for each relationship. The motive score was derived by taking the mean score of all six worsening items within a given relationship from the mean score of all six improving items. The resource score was similarly calculated by taking the mean score of all six behavioral items within a given relationship from the mean score of the six cognitive items. In the second step, the resulting scores on the dimensions of resource and motive for each relationship were treated as Cartesian coordinates (*x*, *y*) from which polar coordinates (*r*, Θ) were calculated (see Figure 2), so that each relationship could be represented as a vector with Θ in radians. In the final step, a single spin score for each participant was computed. Conceptually, spin is the standard deviation of the values of Θ across the relationships, but because observations were vectors rather than scalars, we used Mardia’s (1972) method to calculate the standard deviation (see Moskowitz and Zuroff, 2004, for a detailed description). In brief, the circular variance (CVar) and the circular standard deviation (spin) measure the variability of the individual vectors around the circular mean angle. *M*cos is the mean of the cosines from the angles of those vectors and *M*sin is the mean of the sines. CVar ranges from 0 to 1 and is calculated as [1− √(*M*cos^2^ + *M*sin^2^)]. Spin ranges from 0 to ∞ and is calculated as √ (−2log_e_(1−CVar). Because the resulting spin variable was positively skewed, we used an inverse transformation [calculated as 1−1/(1−spin)] in our analyses.

#### Relationship closeness

Participants were asked to rate the closeness of each relationship they reported on, using the Inclusion of Other in the Self measure (Aron et al., 1992). Aron and colleagues’ measure presents participants with a series of seven pictures each comprising two circles, one representing the “self” and one representing the specified “other” that the participant has chosen to respond about. The first picture has the two circles completely separate and in each successive picture the two circles increasingly overlap. Participants select which picture best describes their relationship and receive a score between 1 (lowest closeness) and 7 (highest closeness). The mean score across all relationships participants reported on was used as an overall index of relationship closeness.

#### Well-being

Two indicators of participants’ well-being were included in the survey. The first was a six-item measure used to assess participants’ moods over the past 4 weeks. Each item was a mood state selected from the UWIST checklist (Matthews et al., 1990) to represent each end of three key dimensions of affect: hedonic tone (“Happy” and “Gloomy”); tense arousal (“Anxious” and “Calm”); and energetic arousal (“Energetic” and “Sluggish”). Negative items were reverse coded so that mean scores represented positive mood. Participants indicated the extent to which they had felt each state over the previous 4 weeks on a seven-point scale from “Not at all” to “A great extent” (α = 0.74). The second indicator was a measure of emotional exhaustion. This measure comprised the four highest loading items from the emotional exhaustion subscale of the Maslach Burnout Inventory (Maslach and Jackson, 1981). For this scale, participants were asked how often they had experienced indicators of emotional exhaustion (e.g., “I felt emotionally drained”) over the past 4 weeks, responding on a five-point scale ranging from “Never” to “All of the time” (α = 0.89).

#### Empathy

The empathic traits of empathic concern and perspective taking were both measured using subscales from Davis’s (1983) Interpersonal Reactivity Index. Both subscales include seven items, for example “I often have tender concerned feelings for people less fortunate then me” for empathic concern (α = 0.74) and “I believe that there are two sides to every question and try to look at them both” for perspective taking (α = 0.72). Participants were required to indicate how well each item described them, on a five-point scale ranging from “does not describe me well” to “describes me very well.”

#### Attachment style

Avoidant and anxious attachment styles were assessed using Brennan et al.’s (1998) Experiences in Close Relationships measure. Measures of attachment style typically ask about people’s relationships with either romantic partners or their parents, with the expectation that this represents a stable underlying pattern of attachment style that will be predictive of their behavior in other relationships, for instance those at work (e.g., Hazan and Shaver, 1990). Brennan and colleagues’ scale asks participants about how they feel and behave in romantic relationships, referring to people’s romantic relationships in general, not just their current romantic relationship (if they have one). There are 36 items in total, 18 of which form the avoidant attachment subscale (e.g., “I prefer not to show a partner how I feel deep down”; α = 0.92), and 18 of which form the anxious attachment subscale (e.g., “I worry about being abandoned”; α = 0.93). Participants indicate how much they agree or disagree with each statement on a seven-point scale ranging from “strongly disagree” to “strongly agree.”

#### Control variables

We measured several variables to serve as controls in our analyses to help rule out possible alternative explanations. Specifically, we controlled for the age and gender of the participant, which might have been related to the outcomes of interest (e.g., relationship closeness, well-being). In addition, we controlled for variability in the gender of the relationship partner (calculated as the standard deviation of the gender of all relationship partners each participant reported on), because it is possible that people use different types of interpersonal emotion regulation strategies toward males and females, which could conflate our results. For a similar reason, we controlled for the number of relationships that participants had reported about (two or three), as higher variability would be expected when reporting on more relationships. Finally, we controlled for the mean amount of interpersonal emotion regulation used across all relationships (calculated as the average of all 12 strategies across all relationships reported on), to ensure that any observed relationships were uniquely relating to interpersonal emotion regulation variability rather than simply the amount of regulation used.

## Results

Mean levels of the use of each type of interpersonal emotion regulation strategy are shown in Table 2. Repeated measures ANOVAs on the sample who had completed data about all three relationships, using relationship type (romantic, friend or relative, work) as the repeated measures factor and mean strategy use scores as dependent variables, revealed significant differences in the use of each of the four main strategy types between the relationships we studied (*F*s ranged between 128.25 and 704.73, *p*s < 0.01). Inspection of the mean scores suggests that all strategy types were used most often within romantic relationships and least often within work relationships. Thus, across the sample as a whole, there was between-relationship variation in the use of interpersonal emotion regulation.

**Table 2:** **Mean use of interpersonal emotion regulation strategies in different relationships**.

	Romantic relationship	Friend or relative	Work relationship	Mean strategy use across relationships
Cognitive improving	3.79	3.49	2.73	3.34
Behavioral improving	3.95	3.43	2.51	3.30
Cognitive worsening	1.76	1.32	1.20	1.43
Behavioral worsening	1.58	1.25	1.21	1.35
Mean use of interpersonal emotion regulation	2.77	2.38	1.92	2.35

**N* = 663, which is the sample completing interpersonal emotion regulation strategies across all three relationships*.

The focus of the current study, however, was on between-relationship variation at the individual-level, operationalized as a person’s level of “spin.” Means, standard deviations, and correlations between spin and the other variables are displayed in Table 3. Correlations involving the main study variables were in line with the view of intra-individual variability as maladaptive. With respect to our control variables, spin was not related to participants’ gender (*r* = −0.04, *p* = 0.14) or age (*r* = 0.03, *p* = 0.23), but was positively related to both the variability in relationship partners’ gender (*r* = 0.06, *p* < 0.05) and the number of relationships reported on (*r* = 0.14, *p* < 0.01).

**Table 3:** **Means, standard deviations, and correlations between main study variables**.

	Mean	SD	1	2	3	4	5	6	7	8	9	10	11	12
1. Gender	–	–												
2. Age	30.96	12.08	−0.06*	–										
3. Variability in gender of partner	0.46	0.28	0.08**	0.03	–									
4. Number of relationships reported	–	–	−0.02	0.17**	0.22**	–								
5. Mean interpersonal emotion regulation	2.43	0.46	0.11*	−0.26**	−0.03	−0.18**	–							
6. Relationship closeness	3.93	1.35	0.06*	−0.26**	<0.01	−0.10**	0.40**	–						
7. Positive mood	3.50	1.10	−0.03	0.14**	−0.05	0.03	−0.02	0.04	–					
8. Emotional exhaustion	2.55	1.01	0.04	−0.18**	0.01	−0.01	0.18**	0.05	−0.53**	–				
9. Empathic concern	3.95	0.61	0.21**	0.03	0.02	0.03	0.13*	0.13*	0.07	0.02	–			
10. Perspective taking	3.51	0.65	0.10	0.25**	0.06	0.04	0.07	<0.01	0.10	0.04	0.42**	–		
11. Avoidant attachment	2.78	1.08	−0.02	0.05	−0.09	−0.27**	−0.06	−0.30**	−0.14*	0.22**	–	–	–	
12. Anxious attachment	3.61	1.29	0.20**	−0.30**	0.02	−0.22**	0.17**	0.03	−0.44**	0.35**	–	–	0.16**	–
13. Spin (inverse transformation)	0.16	0.15	−0.04	0.03	0.06*	0.14**	−0.13**	−0.19**	−0.11**	0.09**	−0.17*	−0.13*	0.04	0.14*

*N* ranges from 228 (for analyses involving empathy), through 273 (for analyses involving attachment), to 1211 for all other analyses. Gender was coded as 1 = female and 0 = male. *p < 0.05, **p < 0.01.

Spin was also negatively related to mean levels of interpersonal emotion regulation across the relationships (*r* = −0.13, *p* < 0.01), signifying that people with high spin are not simply those who use more of all strategies; rather, it is a reflection of the extent of dispersion across relationships. Further exploratory analyses revealed that spin was negatively related to the use of cognitive improving strategies (*r* = −0.42, *p* < 0.01) and behavioral improving strategies (*r* = −0.41, *p* < 0.01), and positively related to the use of cognitive worsening strategies (*r* = 0.38, *p* < 0.01) and behavioral worsening strategies (*r* = 0.48, *p* < 0.01).

Regression results further demonstrate that the observed relationships between spin and the main study variables held after controlling for participant age and gender, variation in partner gender, number of relationships reported, and mean levels of interpersonal emotion regulation. With regard to relationship quality and psychological well-being, regression analyses, shown in Table 4, indicate that spin was negatively related to the closeness of relationships (β = −0.14, *p* < 0.01) and positive mood (β = −0.11, *p* < 0.01), and positively related to emotional exhaustion (β = 0.11, *p* < 0.01).

**Table 4:** **Regression of spin onto relationship closeness, positive mood, and emotional exhaustion**.

	Relationship closeness		Positive mood		Emotional exhaustion	
	β	*t*	β	*t*	β	*t*
Gender	0.01	0.20	−0.02	−0.74	0.02	0.57
Age	−0.17	−6.32**	0.13	3.83**	−0.14	−4.24**
Variability in gender of partner	0.02	0.91	−0.06	−1.60	<0.01	−0.01
Number of relationships reported	0.01	0.30	0.05	1.34	−0.02	−0.44
Mean interpersonal emotion regulation	0.34	12.72**	−0.01	−0.20	0.16	4.89**
Spin	−0.14	−5.41**	−0.11	−3.21**	0.11	3.31**

*N* = 1211. Gender was coded as 1 = female and 0 = male. *p < 0.05, **p < 0.01.

With respect to the individual difference predictors of spin, we ran our regression analyses separately for each predictor so that the effects of each predictor would not be conflated (empathic concern and perspective taking were correlated, as were anxious and avoidant attachment; see Table 3). The results, shown in Table 5, indicate that over and above the control variables, the functional traits we studied were both positively related to spin (empathic concern β = −0.17, *p* < 0.05; perspective taking β = −0.15, *p* < 0.05), while the dysfunctional trait of anxious attachment negatively predicted spin (β = 0.21, *p* < 0.01). Only avoidant attachment was not related to spin (β = 0.09, *p* = 0.14).

**Table 5:** **Regression of individual difference variables onto spin**.

	Empathic concern		Perspective taking		Avoidant attachment		Anxious attachment	
	β	*t*	β	*t*	β	*t*	β	*t*
Gender	0.07	0.98	0.04	0.62	−0.05	−0.83	−0.09	−1.44
Age	0.04	0.61	0.08	1.04	−0.04	−0.72	0.02	0.27
Variability in gender of partner	0.04	0.61	0.05	0.70	−0.01	−0.11	−0.02	−0.33
Number of relationships reported	0.03	0.45	0.03	0.36	0.19	2.84**	0.19	3.02**
Mean interpersonal emotion regulation	−0.12	−1.72	−0.11	−1.58	<0.01	0.02	−0.03	−0.42
Empathic concern	−0.17	−2.58*						
Perspective taking			−0.15	−2.22				
Avoidant attachment					0.09	1.48		
Anxious attachment							0.21	3.25**

*N* = 228 for analyses involving empathy, *N* = 273 for analyses involving attachment. Gender was coded as 1 = female and 0 = male. *p < 0.05, **p < 0.01.

### Supplementary analyses

Because spin was differentially related to mean use of different types of strategies, we also conducted exploratory analyses to investigate whether variability in different types of interpersonal emotion regulation related to the main study variables. To assess such variability, we calculated four measures of “flux” to assess the standard deviation in the use across different relationships of improving strategies, worsening strategies, cognitive strategies, and behavioral strategies (Moskowitz and Zuroff, 2004). Equivalent “spin” scores cannot be calculated as spin quantifies variability across two dimensions.

The results in Table 6 indicate that variability in the use of all types of strategies, with the exception of worsening strategies, was negatively related to relationship closeness, while variability in the use of worsening strategies and cognitive strategies was negatively related to positive mood and positively related to emotional exhaustion. Regarding the individual differences, empathic concern, and perspective taking were unrelated to variability in the use of any single type of strategy. In contrast, avoidant attachment was related to variability in the use of improving strategies and anxious attachment was related to variability in the use of worsening strategies and cognitive strategies. These results verify the notion that higher variability may be maladaptive, and further suggest that varying one’s use of certain types of strategies may be more maladaptive for some outcomes than others.

**Table 6:** **Correlations between variability in the use of different types of strategies and main study variables**.

	Flux in improving strategies	Flux in worsening strategies	Flux in cognitive strategies	Flux in behavioral strategies
Flux in improving strategies	–			
Flux in worsening strategies	0.07**	–		
Flux in cognitive strategies	0.62**	0.37**	–	
Flux in behavioral strategies	0.65**	0.37**	0.63**	–
Relationship closeness	−0.14**	−0.01	−0.10**	−0.14**
Positive mood	<0.01	−0.17**	−0.09**	−0.03
Emotional exhaustion	0.03	0.20**	0.08**	0.06
Empathic concern	0.03	0.03	0.03	0.03
Perspective taking	0.04	−0.07	0.01	0.03
Avoidant attachment	0.13*	0.05	−0.05	−0.03
Anxious attachment	0.06	0.28**	0.22**	0.09

*N* ranges from 228 (for analyses involving empathy), through 273 (for analyses involving attachment), to 1211 for all other analyses. *p < 0.05, **p < 0.01.

## Discussion

The regulation of others’ feelings is a common feature of most of the important relationships people have, e.g., those with romantic partners, friends or family members, and people at work. Our findings indicate that variability in a person’s interpersonal emotion regulation strategy use across these different relationships, as indicated by a person’s level of interpersonal “spin,” is associated with higher emotional exhaustion and lower positive mood and relationship closeness. Moreover, high anxious attachment style, low empathic concern, and low perspective taking were associated with higher levels of spin. These findings suggest that, in line with previous research on spin, high variability in the use of interpersonal emotion regulation can be considered maladaptive for both personal and social functioning (Moskowitz and Zuroff, 2004).

Our findings are consistent with theoretical arguments that high variability in interpersonal emotion regulation is a sign of heightened reactivity to the influence of situations (Erickson et al., 2009). The result of this heightened reactivity is an inability to maintain consistency in interactions, and interactions becoming more effortful and demanding yet less successful in terms of goal-pursuit. As such, interpersonal emotion regulation variability can be considered poorly controlled (Moskowitz and Zuroff, 2004) and therefore maladaptive for both personal and social functioning.

A potential alternative explanation is that our findings were strongly influenced by the use of strategies to worsen others’ emotions. Strategies to worsen others’ emotions have previously been linked to negative outcomes (e.g., poor well-being; Niven et al., 2012b) and in the present study mean use of worsening strategies was associated with spin. It could therefore be the case that people who exhibited greater overall variability in their use of strategies were those who engaged more in worsening strategies, which are likely less adaptive. Our supplementary analyses, however, highlighted that while variability in the use of affect-worsening strategies was particularly maladaptive for personal well-being, it was not so maladaptive for social functioning, showing no association with the closeness of relationships. In contrast, higher variability in the use of other strategy types (affect-improving, cognitive, and behavioral) was associated with lower relationship closeness. Thus, the maladaptive nature of variability is unlikely to be driven purely by use of or variability in strategies to worsen others’ feelings.

Against expectations, we did not observe a relationship between avoidant attachment style and interpersonal spin. We had anticipated this relationship because avoidant attachment style is typically considered dysfunctional (people with an avoidant attachment style tend to have poorer quality relationships, characterized by anger, hostility, and distress; Mikulincer, 1998; Shaver et al., 2005), and prior research has suggested that interpersonal spin is connected to other traits and disorders that are maladaptive, such as neuroticism and borderline personality disorder (e.g., Moskowitz and Zuroff, 2004; Russell et al., 2007). One possible explanation for our incongruous finding is that because people with avoidant attachment style have a strong need for maintain independence and emotional distance from others, they may be similarly disengaged within all their relationships. However, it is worth noting that we did find a relationship between avoidant attachment style and variability across relationships (flux) in the use of improving strategies.

The present study makes a key contribution to research on interpersonal emotion regulation. To date, most studies of this process have focused on exploring the divergent effects of different strategies used to regulate others’ emotions (e.g., Niven et al., 2007, 2012b), with little consideration of the notion that people may vary the strategies they use in different relationships. The present study therefore represents the first attempt to document differences in interpersonal emotion regulation use between relationships, and the first to investigate whether greater variation in strategy use is functional or dysfunctional for people’s well-being and relationship development.

A second key contribution of this research is with regard to studies of intra-individual variability. Previous studies have examined variability of interpersonal behavior and core affect, using the framework of spin (e.g., Moskowitz and Zuroff, 2004; Kuppens et al., 2007; Côté et al., 2011). However, the present study is the first to apply the ideas from these fields to the specific area of interpersonal emotion regulation. Similarly, it is the first to consider the association between spin and traits such as empathy and attachment styles, as prior studies have focused on self-esteem and the Big-5 traits. That our findings are in line with those reported in prior intra-individual variability research adds weight to the body of evidence suggesting that high variability might be maladaptive and an indicator of instability and behavioral liability.

Nonetheless, there are some important limitations of the present study. First, our results are all based on self-reported cross-sectional data, which could be subject to biases, including social desirability. However, the validation of our self-reported interpersonal emotion regulation data against relationship partners’ reports, along with the fact that the key variable of interest, spin, was an indicator of variability across relationships rather than a mean score, gives us confidence that such biases have not unduly affected our findings. The direction of causality also cannot be stated with certainty, and thus future longitudinal research is needed on this subject. Second, due to a desire not to overload participants in the study, we only studied three types of relationships (romantic, friend or family, work), whereas interpersonal emotion regulation may be used in many other relational contexts (e.g., towards support group members, teammates in sports, or even strangers; Cahill and Eggleston, 1994; Thoits, 1996; Friesen et al., 2011), which might show meaningful variation. Third, unlike some other studies that have used daily reports of interactions to calculate interpersonal spin (e.g., Moskowitz and Zuroff, 2004; Côté et al., 2011), we calculated spin based on responses to a one-off survey, asking about people’s use of interpersonal emotion regulation in different relationships. This had the clear advantage of allowing us to equally represent each different type of relationship of interest in our spin score (in diary studies, respondents might, for example, report only interactions with their romantic partner, meaning that other types of relationships are not well-represented). However, an important disadvantage of this approach is that intra-individual variability over time within the same relationship is not captured. Future studies of variability in interpersonal emotion regulation could therefore use a daily diary method and extend the range of relationships participants report on.

## Conflict of Interest Statement

The authors declare that the research was conducted in the absence of any commercial or financial relationships that could be construed as a potential conflict of interest.
